# Crystal structure and Hirshfeld surface analysis of new polymorph of racemic 2-phenyl­butyramide

**DOI:** 10.1107/S2056989019007011

**Published:** 2019-05-21

**Authors:** Sergei Rigin, Beatrice Armijo, Arcadius V. Krivoshein, Marina Fonari, Tatiana Timofeeva

**Affiliations:** a Department of Chemistry, New Mexico Highlands University, Las Vegas, New Mexico, 87701, USA; b Department of Physical & Applied Sciences, University of Houston – Clear Lake, 2700 Bay Area Boulevard, Houston, TX 77058, USA; c Institute of Applied Physics, Academy str., 5 MD2028, Chisinau, Moldova

**Keywords:** crystal structure, racemate, 2-phenyl­butyramide, hydrogen bonds, Hirshfeld surface analysis

## Abstract

A new polymorph of the title compound, C_10_H_13_NO, was obtained by recrystallization of the commercial product from a water/ethanol mixture (1:1 *v*/*v*). Crystals of the previously reported racemic and homochiral forms of 2-phenyl­butyramide were grown from water–aceto­nitrile solution in 1:1 volume ratio [Khrustalev *et al.* (2014 ▸). *Cryst. Growth Des.* **14**, 3360–3369]. While the previously reported racemic and enanti­opure forms of the title compound adopt very similar supra­molecular structures (hydrogen-bonded ribbons), the new racemic polymorph is stabilized by a single N—H⋯O hydrogen bond that links mol­ecules into chains along the *c*-axis direction with an anti­parallel (centrosymmetric) packing in the crystal. Hirshfeld mol­ecular surface analysis was employed to compare the inter­molecular inter­actions in the polymorphs of the title compound.

## Abstract 2

The crystal structure of new polymorph is reported in which an N—H⋯O hydrogen bond links mol­ecules into chains with anti­parallel (centrosymmetric) packing.

## Chemical context   

Many drugs exist in several polymorphic modifications (Bernstein, 2011 ▸; Brittain, 2009 ▸). For example, a second polymorph, II, was reported (Vishweshwar *et al.*, 2005 ▸) in 2005 ▸ for aspirin, one of the most widely consumed medications; this was similar in structure to the original form I (Wheatley, 1964 ▸) and was widely discussed (Bond *et al.*, 2007 ▸, 2011 ▸). The third ambient polymorph of aspirin, crystallized from the melt, was described recently using a combination of X-ray powder diffraction analysis and crystal structure prediction algorithms (Shtukenberg *et al.*, 2017 ▸). Paracetamol, one of the most frequently used anti­pyretic and analgesic drugs, is known in three crystal modifications: a monoclinic (form I) (Haisa *et al.*, 1976 ▸), an ortho­rhom­bic (form II) (Haisa *et al.*, 1974 ▸), and an unstable phase (form III), which can only be stabilized under certain conditions (Burger & Ramberger, 1979 ▸). The non-steroidal anti-inflammatory drug mefenamic acid is known to exist as dimorphs (I and II) and a metastable polymorph obtained during co-crystallization experiments (SeethaLekshmi & Guru Row, 2012 ▸). The existence of three different polymorphic forms has been reported for anhydrous carbamazepine, an anti­convulsant drug (Rustichelli *et al.*, 2000 ▸). We previously reported (Khrustalev *et al.*, 2014 ▸) two polymorphs of α-methyl-α-phenyl­succinimide (3-methyl-3-phenyl­pyrrol­idine-2,5-dione), the *N*-de­methyl­ated metabolite of the anti­convulsant methsuximide. Herein, we report on the crystal structure and the Hirshfeld surface analysis of a new polymorph of the title compound, obtained by recrystallization of the commercial product (Alfa Aesar, stock No. A18501) from a water–ethanol (1:1) solution. Crystals of the previously reported racemic and homochiral forms of 2-phenyl­butyramide were grown from water–aceto­nitrile solution in a 1:1 volume ratio (Khrustalev *et al.*, 2014 ▸).
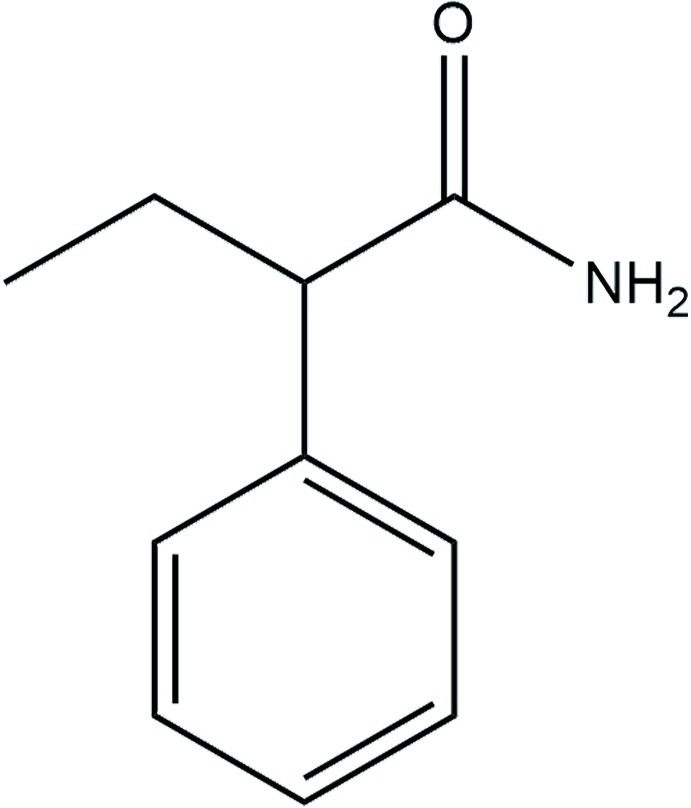


## Structural commentary   

**Figure 1:**
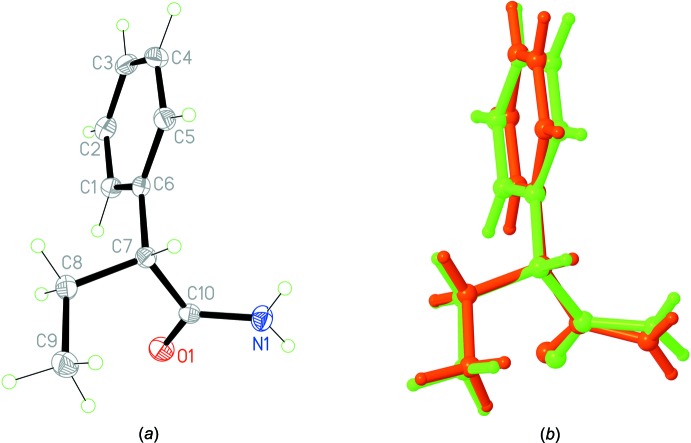
(*a*) View of the mol­ecular structure of the title *rac*-2 polymorph with the atom labelling. Displacement ellipsoids are drawn at the 50% probability level. (*b*) Superposition of the *rac*-1 (red) and *rac*-2 (green) polymorphs.

A view of the mol­ecule of the new polymorph (henceforth referred to as *rac-*2) is illustrated in Fig. 1 ▸ *a*. In the mol­ecule, the rotation of the amide group around the C7—C10 bond is characterized by an N1—C10—C7—C8 torsion angle of −141.14 (9)°, thus corresponding in conformation to the previously reported polymorph (further notated as *rac*-1, space group *C*2/*c*), where the torsion angle is −130.06 (9)°, and one of two conformers in the *R*-enanti­omer [space group *P*1, torsion angle = −144.08 (13)°; Khrustalev *et al.*, 2014 ▸). The overlay diagram for the two racemic forms shown in Fig. 1 ▸ *b* shows the almost perfect fit (r.m.s. deviation = 0.263 Å). The bond lengths in the mol­ecule are in line with those of reported analogues (CSD version 5.40, last update November 2018; Groom *et al.*, 2016 ▸).

## Supra­molecular features   

**Figure 2:**
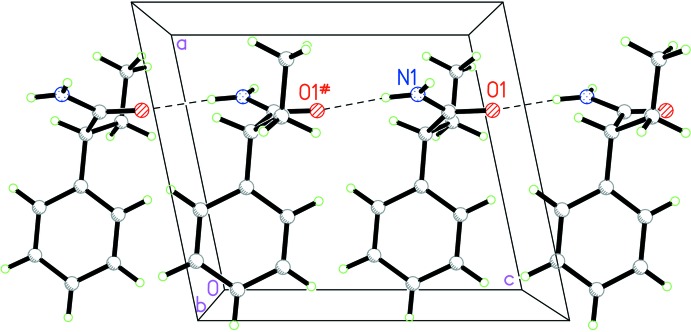
View of a hydrogen-bonded chain.

**Figure 3:**
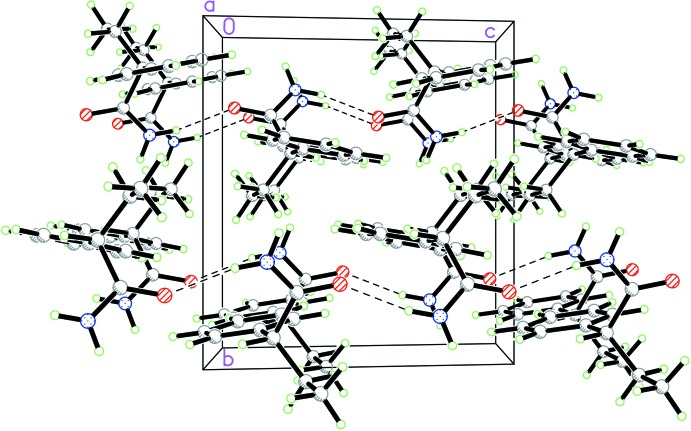
The crystal packing of the *rac*-2 polymorph.

Mol­ecule of the title compound contain one amino group as a potential double hydrogen-bond donor and one carbonyl group capable of acting as a multiple hydrogen-bond acceptor. Contrary to the previously reported *rac*-1 and two enanti­omeric forms (Khrustalev *et al.*, 2014 ▸), where the amino group acted as double hydrogen-bond donor while the carbonyl oxygen atom acted as a double hydrogen-bond acceptor being involved in two N–H⋯O hydrogen bonds leading to the formation of supra­molecular ribbons, in *rac*-2 only one hydrogen atom of the amino group is involved in a single N—H⋯O hydrogen bond (Table 1 ▸). This hydrogen bond links mol­ecules related by the glide plane into chains along the *c*-axis direction (Fig. 2 ▸). The packing of the chains obeys inversion symmetry with only van der Waals contacts between the chains (Fig. 3 ▸). In spite of the fewer number of strong directed inter­molecular inter­actions in the crystal, the structure of *rac-*2 is characterized by a more effective crystal packing of the single chains, compared to the packing of ribbons in *rac-*1 and in the enanti­omers, which follows from the higher value of the crystal density (calculated as 1.227 g cm^−3^; Table 2 ▸) compared with values of 1.160 g cm^−3^ for *rac*-1 and 1.188 g cm^−3^ and 1.189 g cm^−3^ for the *R*- and *S*-enanti­omers (Khrustalev *et al.*, 2014 ▸).

## Hirshfeld surface analysis and calculation of crystal lattice energies   

**Figure 4:**
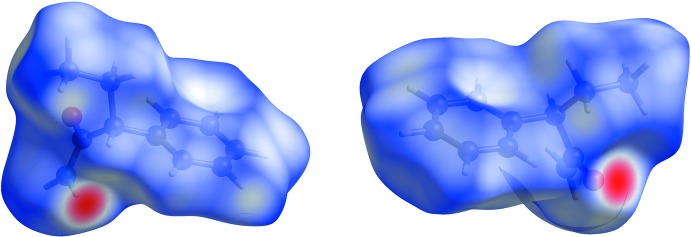
Hirshfeld surface for the *rac*-2 polymorph plotted over *d*_norm_ in the range −0.4994 to 1.0567 a.u.

**Figure 6:**
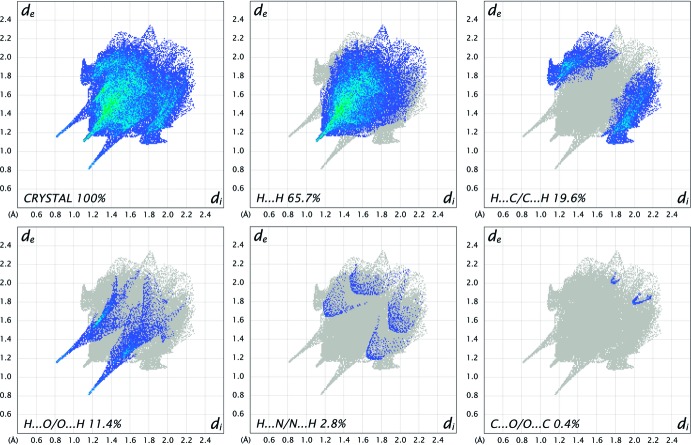
The full two-dimensional fingerprint plots for the *rac*-2 polymorph, showing all inter­actions, and delineated into H⋯H, H⋯C/C⋯H, H⋯O/O⋯H, H⋯N/N⋯H, C⋯O/O⋯C inter­actions. The *d*_i_ and *d*_e_ values are the closest inter­nal and external distances (in Å) from given points on the Hirshfeld surface.

**Figure 7:**
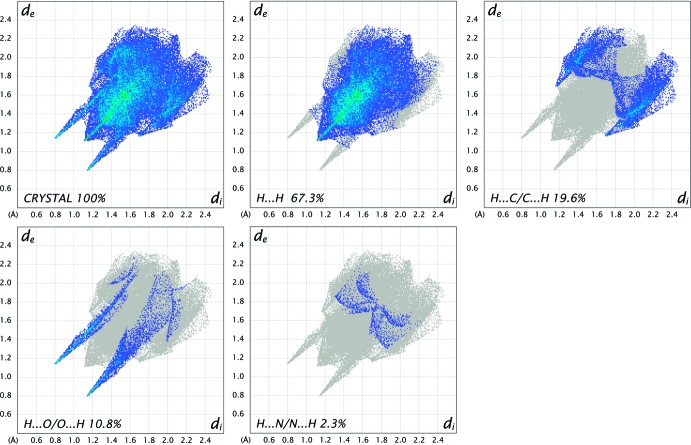
The full two-dimensional fingerprint plots for the *rac*-1 polymorph, showing all inter­actions, and delineated into H⋯H, H⋯C/C⋯H, H⋯O/O⋯H and H⋯N/N⋯H inter­actions. The *d*_i_ and *d*_e_ values are the closest inter­nal and external distances (in Å) from given points on the Hirshfeld surface.

*Crystal Explorer* (Wolff *et al.*, 2012 ▸) was used to generate the Hirshfeld surfaces (Hirshfeld, 1977 ▸). The total *d*_norm_ surfaces for polymorphs *rac*-2 and *rac*-1 are shown in Figs. 4 ▸ and 5 ▸, respectively, in which the red spots correspond to the most significant N—H⋯O inter­actions in the crystal (Table 1 ▸). The surface diagram unambiguously shows that there are fewer active binding sites in *rac-*2 in comparison to *rac-*1. The two-dimensional fingerprint plots from the Hirshfeld surface analysis (Spackman & Jayatilaka, 2009 ▸) allows the inter­molecular inter­actions to be analysed in detail and for even rather subtle differences between polymorphic systems to be qu­anti­fied (Bernstein, 2011 ▸). The two-dimensional fingerprint plots for *rac-2* and *rac-1* are shown in Figs. 6 ▸ and 7 ▸, respectively. They clearly indicate the different distribution of inter­actions for a single mol­ecule in the two structures. Decomposition of the full fingerprint plot for *rac*-2 shows five principle types of inter­actions that include H⋯H, H⋯C/C⋯H, H⋯O/O⋯H, H⋯N/N⋯H, and C⋯O/O⋯C contacts in decreasing order (Fig. 6 ▸). For the *rac*-1 polymorph, the set includes only four types of inter­actions, *viz*. H⋯H, H⋯C/C⋯H, H⋯O/O⋯H and H⋯N/N⋯H contacts (Fig. 7 ▸). The predominant inter­actions in both cases are H⋯H, constituting 65.7% in *rac*-2 and 67.3% in *rac-*1. With a significantly less contribution, the next most important inter­actions are H⋯C/C⋯H, contributing 19.6% in both cases, and being slightly asymmetric in shape in favour of (inter­nal)C⋯H(external) contacts for both polymorphs. The directed H⋯O/O⋯H contacts constitute 11.4% for *rac*-2 and 10.8% for *rac*-1, with slight a asymmetry in favour of (inter­nal)O⋯H(external) contacts for both polymorphs.

The Hirshfeld surface analysis confirms the decisive role of H-contacts that include hydrogen bonding and van der Waals inter­actions in the crystal packing. The crystal-lattice energies (Table 2 ▸) were calculated from the atomic coordinates obtained in the single-crystal X-ray diffraction experiments using the atom–atom force field with subdivision of the inter­action energies into Coulombic, polarization, London dispersion, and Pauli repulsion components (*AA-CLP*; Gavezzotti, 2011 ▸, 2013 ▸) implemented in the *CLP-PIXEL* computer program package (version 3.0, available from www.angelogavezzotti.it). These show that the *rac*-2 polymorph is more stable in terms of two criteria: total crystal energy and crystal density.

## Database survey   

The Cambridge Structural Database (CSD version 5.40, last update November 2018; Groom *et al.*, 2016 ▸) includes crystallographic data for the *R*- and *S*-enanti­omers of 2-phenyl­butyramide (VOQGUF and VOQHAM, space group *P*1; Khrustalev *et al.*, 2014 ▸) and the racemic form (VOQHEQ, space group *C*2/*c*; Khrustalev *et al.*, 2014 ▸). As mentioned above, the conformations of two *rac-*polymorphs are quite similar, while the crystal packing differs significantly with more efficient crystal packing for the *rac-*2 polymorph reported here.

## Crystallization   

Crystals were obtain by the slow evaporation approach. 0.5 g of 2-phenyl­butyramide (Alfa Aesar, stock No. A18501) were dissolved with extensive vortexing in 3 mL of a water/ethanol mixture (1:1 *v*/*v*) and left at room temperature (293–295 K) for six weeks. Block-shaped crystals formed on the walls of the vessel.

## Refinement   

Crystal data, data collection and structure refinement details are summarized in Table 3 ▸.

## Supplementary Material

Crystal structure: contains datablock(s) I. DOI: 10.1107/S2056989019007011/yk2123sup1.cif

Structure factors: contains datablock(s) I. DOI: 10.1107/S2056989019007011/yk2123Isup2.hkl

Supporting information file. DOI: 10.1107/S2056989019007011/yk2123Isup3.cml

CCDC reference: 1916098

Additional supporting information:  crystallographic information; 3D view; checkCIF report

## Figures and Tables

**Figure 5:**
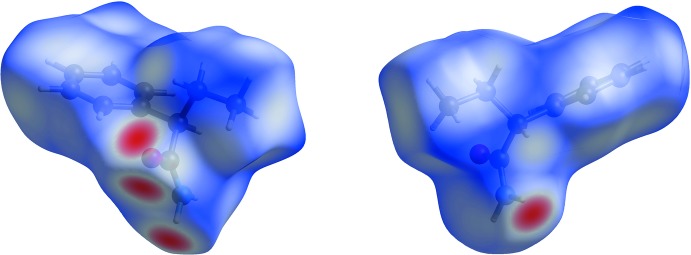
Hirshfeld surface for the *rac*-1 polymorph plotted over *d*_norm_ in the range −0.5239 to 1.3882 a.u.

**Table 1:** Hydrogen-bond geometry (Å, °)

*D*—H⋯*A*	*D*—H	H⋯*A*	*D*⋯*A*	*D*—H⋯*A*
N1—H1*B*⋯O1^i^	0.895 (15)	2.071 (15)	2.9533 (13)	168.4 (13)

Symmetry code: (i) 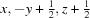.

**Table 2:** Crystal lattice energies (kJ mol^−1^) for racemic 2-phenyl­butyramide polymorphs computed using *AA-CLP* software

Polymorph	*E*_electrostatic_	*E*_polarization_	*E*_dispersion_	*E*_exchange-repulsion_	*E*_total_	Crystal density (g cm^−3^)
*rac*-1	−35.6*^*a*^*	−27.6*^*a*^*	−100.4*^*a*^*	55.0*^*a*^*	−108.6*^*a*^*	1.160*^*b*^*
*rac*-2	−33.9	−28.3	−107.9	53.8	−116.3	1.227

Notes: ^*a*^from Krivoshein *et al.* (2018 ▸); ^*b*^from Khrustalev *et al.* (2014 ▸).

**Table 3:** Experimental details

Crystal data	
Chemical formula	C_10_H_13_NO
*M*_r_	163.21
Crystal system, space group	Monoclinic, *P*2_1_/*c*
Temperature (K)	100
*a*, *b*, *c* (Å)	8.575 (2), 10.746 (3), 9.798 (3)
β (°)	101.811 (3)
*V* (Å^3^)	883.8 (4)
*Z*	4
Radiation type	Mo *K*α
μ (mm^−1^)	0.08
Crystal size (mm)	0.15 × 0.1 × 0.1

Data collection	
Diffractometer	Bruker APEXII CCD
Absorption correction	Multi-scan (*SADABS*; Bruker, 2004 ▸)
*T*_min_, *T*_max_	0.674, 0.746
No. of measured, independent and observed [*I* > 2σ(*I*)] reflections	9961, 2240, 1914
*R*_int_	0.038
(sin θ/λ)_max_ (Å^−1^)	0.671

Refinement	
*R*[*F* ^2^ > 2σ(*F* ^2^)], *wR*(*F* ^2^), *S*	0.040, 0.110, 1.08
No. of reflections	2240
No. of parameters	161
H-atom treatment	All H-atom parameters refined
Δρ_max_, Δρ_min_ (e Å^−3^)	0.31, −0.27

Computer programs: *APEX2* and *SAINT* (Bruker, 2004 ▸), *SHELXT* (Sheldrick, 2015*a* ▸), *SHELXL* (Sheldrick, 2015*b* ▸)and *OLEX2* (Dolomanov *et al.*, 2009 ▸).

## supplementary crystallographic information

### Crystal data

**Table:**

C_10_H_13_NO	*F*(000) = 352
*M**_r_* = 163.21	*D*_x_ = 1.227 Mg m^−^^3^
Monoclinic, *P*2_1_/*c*	Mo *K*α radiation, λ = 0.71073 Å
*a* = 8.575 (2) Å	Cell parameters from 3598 reflections
*b* = 10.746 (3) Å	θ = 2.4–30.1°
*c* = 9.798 (3) Å	µ = 0.08 mm^−^^1^
β = 101.811 (3)°	*T* = 100 K
*V* = 883.8 (4) Å^3^	Block, colourless
*Z* = 4	0.15 × 0.1 × 0.1 mm

### Data collection

**Table:**

Bruker APEXII CCD diffractometer	1914 reflections with *I* > 2σ(*I*)
φ and ω scans	*R*_int_ = 0.038
Absorption correction: multi-scan (SADABS; Bruker, 2004)	θ_max_ = 28.5°, θ_min_ = 2.4°
*T*_min_ = 0.674, *T*_max_ = 0.746	*h* = −11→10
9961 measured reflections	*k* = −14→14
2240 independent reflections	*l* = −13→13

### Refinement

**Table:**

Refinement on *F*^2^	Primary atom site location: dual
Least-squares matrix: full	Hydrogen site location: difference Fourier map
*R*[*F*^2^ > 2σ(*F*^2^)] = 0.040	All H-atom parameters refined
*wR*(*F*^2^) = 0.110	*w* = 1/[σ^2^(*F*_o_^2^) + (0.0614*P*)^2^ + 0.1474*P*] where *P* = (*F*_o_^2^ + 2*F*_c_^2^)/3
*S* = 1.08	(Δ/σ)_max_ < 0.001
2240 reflections	Δρ_max_ = 0.31 e Å^−^^3^
161 parameters	Δρ_min_ = −0.27 e Å^−^^3^
0 restraints	

### Special details

**Table:**

Geometry. All esds (except the esd in the dihedral angle between two l.s. planes) are estimated using the full covariance matrix. The cell esds are taken into account individually in the estimation of esds in distances, angles and torsion angles; correlations between esds in cell parameters are only used when they are defined by crystal symmetry. An approximate (isotropic) treatment of cell esds is used for estimating esds involving l.s. planes.

### Fractional atomic coordinates and isotropic or equivalent isotropic displacement parameters (Å^2^)

**Table:**

	*x*	*y*	*z*	*U*_iso_*/*U*_eq_	
O1	0.32919 (9)	0.23775 (7)	0.06137 (7)	0.0216 (2)	
N1	0.27946 (11)	0.17768 (8)	0.26886 (9)	0.0192 (2)	
H1A	0.2469 (17)	0.1033 (15)	0.2342 (15)	0.031 (4)*	
H1B	0.2895 (17)	0.1927 (14)	0.3601 (15)	0.028 (3)*	
C1	0.68011 (12)	0.34374 (9)	0.20614 (10)	0.0179 (2)	
H1	0.6296 (16)	0.3276 (12)	0.1099 (14)	0.021 (3)*	
C2	0.84492 (13)	0.33963 (10)	0.24848 (11)	0.0206 (2)	
H2	0.9097 (17)	0.3192 (13)	0.1809 (14)	0.027 (3)*	
C3	0.91672 (13)	0.36033 (10)	0.38704 (11)	0.0217 (2)	
H3	1.0322 (19)	0.3568 (14)	0.4177 (15)	0.035 (4)*	
C4	0.82161 (13)	0.38440 (10)	0.48320 (11)	0.0219 (2)	
H4	0.8698 (17)	0.3979 (13)	0.5822 (15)	0.027 (3)*	
C5	0.65680 (13)	0.38737 (9)	0.44137 (10)	0.0184 (2)	
H5	0.5908 (15)	0.4038 (12)	0.5099 (13)	0.017 (3)*	
C6	0.58384 (12)	0.36770 (8)	0.30233 (10)	0.0151 (2)	
C7	0.40388 (12)	0.37876 (9)	0.25694 (10)	0.0150 (2)	
H7	0.3586 (15)	0.3913 (12)	0.3424 (13)	0.019 (3)*	
C8	0.35882 (12)	0.49138 (9)	0.16087 (11)	0.0189 (2)	
H8A	0.4084 (18)	0.5661 (13)	0.2142 (15)	0.030 (4)*	
H8B	0.4098 (16)	0.4803 (12)	0.0771 (14)	0.024 (3)*	
C9	0.18019 (13)	0.50974 (11)	0.11476 (12)	0.0226 (2)	
H9A	0.1318 (18)	0.4394 (14)	0.0522 (15)	0.033 (4)*	
H9B	0.1575 (19)	0.5862 (15)	0.0573 (16)	0.039 (4)*	
H9C	0.1253 (18)	0.5153 (14)	0.1957 (16)	0.034 (4)*	
C10	0.33405 (11)	0.25883 (9)	0.18573 (10)	0.0148 (2)	

### Atomic displacement parameters (Å^2^)

**Table:**

	*U*^11^	*U*^22^	*U*^33^	*U*^12^	*U*^13^	*U*^23^
O1	0.0261 (4)	0.0250 (4)	0.0141 (4)	−0.0021 (3)	0.0046 (3)	−0.0035 (3)
N1	0.0241 (5)	0.0158 (4)	0.0184 (4)	−0.0028 (3)	0.0060 (3)	−0.0006 (3)
C1	0.0198 (5)	0.0181 (5)	0.0155 (4)	−0.0020 (4)	0.0026 (4)	−0.0003 (3)
C2	0.0202 (5)	0.0185 (5)	0.0244 (5)	−0.0016 (4)	0.0073 (4)	−0.0004 (4)
C3	0.0168 (5)	0.0182 (5)	0.0279 (5)	−0.0025 (4)	−0.0004 (4)	0.0011 (4)
C4	0.0259 (6)	0.0191 (5)	0.0177 (5)	−0.0025 (4)	−0.0025 (4)	−0.0003 (4)
C5	0.0227 (5)	0.0160 (5)	0.0162 (5)	−0.0006 (4)	0.0034 (4)	−0.0003 (3)
C6	0.0167 (5)	0.0118 (4)	0.0163 (4)	−0.0012 (3)	0.0022 (3)	0.0010 (3)
C7	0.0163 (5)	0.0159 (5)	0.0129 (4)	−0.0007 (3)	0.0035 (3)	−0.0002 (3)
C8	0.0187 (5)	0.0176 (5)	0.0202 (5)	−0.0003 (4)	0.0036 (4)	0.0036 (4)
C9	0.0196 (5)	0.0231 (5)	0.0244 (5)	0.0031 (4)	0.0028 (4)	0.0036 (4)
C10	0.0131 (4)	0.0158 (4)	0.0150 (4)	0.0012 (3)	0.0020 (3)	−0.0003 (3)

### Geometric parameters (Å, º)

**Table:**

O1—C10	1.2316 (12)	C5—H5	0.979 (12)
N1—H1A	0.890 (16)	C5—C6	1.3940 (14)
N1—H1B	0.895 (15)	C6—C7	1.5206 (14)
N1—C10	1.3414 (13)	C7—H7	1.002 (12)
C1—H1	0.970 (13)	C7—C8	1.5333 (14)
C1—C2	1.3898 (15)	C7—C10	1.5282 (13)
C1—C6	1.3982 (14)	C8—H8A	1.004 (14)
C2—H2	0.973 (14)	C8—H8B	1.013 (13)
C2—C3	1.3891 (15)	C8—C9	1.5187 (15)
C3—H3	0.975 (16)	C9—H9A	1.007 (15)
C3—C4	1.3909 (16)	C9—H9B	0.992 (16)
C4—H4	0.984 (14)	C9—H9C	1.004 (15)
C4—C5	1.3890 (15)		
			
H1A—N1—H1B	120.1 (13)	C6—C7—H7	108.0 (7)
C10—N1—H1A	118.2 (9)	C6—C7—C8	110.76 (8)
C10—N1—H1B	121.0 (9)	C6—C7—C10	110.30 (8)
C2—C1—H1	120.6 (8)	C8—C7—H7	108.4 (7)
C2—C1—C6	120.62 (9)	C10—C7—H7	108.2 (7)
C6—C1—H1	118.8 (8)	C10—C7—C8	111.06 (8)
C1—C2—H2	119.4 (8)	C7—C8—H8A	106.5 (8)
C3—C2—C1	120.46 (10)	C7—C8—H8B	107.9 (8)
C3—C2—H2	120.1 (8)	H8A—C8—H8B	108.0 (11)
C2—C3—H3	120.9 (9)	C9—C8—C7	113.41 (8)
C2—C3—C4	119.20 (10)	C9—C8—H8A	110.2 (8)
C4—C3—H3	119.9 (9)	C9—C8—H8B	110.5 (8)
C3—C4—H4	120.6 (8)	C8—C9—H9A	110.4 (8)
C5—C4—C3	120.45 (9)	C8—C9—H9B	110.2 (9)
C5—C4—H4	119.0 (8)	C8—C9—H9C	112.4 (9)
C4—C5—H5	119.9 (7)	H9A—C9—H9B	105.6 (12)
C4—C5—C6	120.73 (9)	H9A—C9—H9C	108.9 (12)
C6—C5—H5	119.4 (7)	H9B—C9—H9C	109.2 (12)
C1—C6—C7	121.41 (9)	O1—C10—N1	122.40 (9)
C5—C6—C1	118.55 (9)	O1—C10—C7	122.52 (9)
C5—C6—C7	119.98 (9)	N1—C10—C7	115.07 (8)
			
C1—C2—C3—C4	0.44 (16)	C5—C6—C7—C8	112.46 (10)
C1—C6—C7—C8	−64.48 (12)	C5—C6—C7—C10	−124.17 (9)
C1—C6—C7—C10	58.89 (11)	C6—C1—C2—C3	−0.55 (16)
C2—C1—C6—C5	0.03 (15)	C6—C7—C8—C9	−178.47 (8)
C2—C1—C6—C7	177.01 (9)	C6—C7—C10—O1	−83.85 (11)
C2—C3—C4—C5	0.18 (15)	C6—C7—C10—N1	95.67 (10)
C3—C4—C5—C6	−0.70 (15)	C8—C7—C10—O1	39.34 (13)
C4—C5—C6—C1	0.59 (15)	C8—C7—C10—N1	−141.14 (9)
C4—C5—C6—C7	−176.43 (9)	C10—C7—C8—C9	58.60 (11)

### Hydrogen-bond geometry (Å, º)

**Table:**

*D*—H···*A*	*D*—H	H···*A*	*D*···*A*	*D*—H···*A*
N1—H1*B*···O1^i^	0.895 (15)	2.071 (15)	2.9533 (13)	168.4 (13)

Symmetry code: (i) *x*, −*y*+1/2, *z*+1/2.
